# The Breast Cancer Screening and Timing of Breast MRI—Experience in a Genetic High-Risk Screening Clinic in a Comprehensive Cancer Center

**DOI:** 10.3390/curroncol29030171

**Published:** 2022-03-19

**Authors:** Xia Wang, Maxine D. Chang, Marie Catherine Lee, Bethany L. Niell

**Affiliations:** 1 GeneHome, Department of Individualized Cancer Management, H. Lee Moffitt Cancer Center & Research Institute, Tampa, FL 33612, USA; maxine.chang@moffitt.org; 2 Department of Breast Oncology, H. Lee Moffitt Cancer Center & Research Institute, Tampa, FL 33612, USA; marie.lee@moffitt.org; 3 Division of Breast Imaging, Department of Diagnostic Imaging, H. Lee Moffitt Cancer Center & Research Institute, Tampa, FL 33612, USA; bethany.niell@moffitt.org

**Keywords:** breast MRI, mammography, high-risk screening, breast cancer, genetic predisposition, *BRCA1*, *BRCA2*, *CDH1*, *PTEN*, *TP53*

## Abstract

For women with genetic risk of breast cancer, the addition of screening breast MRI to mammography has become a standard. The order and interval of annual imaging can be variable among providers. To evaluate the clinical implications related to the timing, we conducted a chart review on a cohort of women (*N* = 276) with high-risk (*BRCA1*, *BRCA2*, *CDH1*, *PTEN* and *TP53*) and moderate high-risk (*ATM* and *CHEK2*) predisposition to breast cancer in a 48-month follow up. The estimated MRI detection rate in the entire group is 1.75% (18 per 1000 MRI tests). For the high-risk group, the estimated rate is 2.98% (30 per 1000 MRI tests). Many women discovered their genetic risk at an age much older (average age of the high-risk group was 48 years) than the age recommended to initiate enhanced screening (age 20 to 25 years). In total, 4 of the 11 primary breast cancers detected were identified by screening MRI within the first month after initial visit, which were not detected by previous mammography, suggesting the benefit of initiating MRI immediately after the discovery of genetic risk. Breast screening findings for women with Lynch syndrome and neurofibromatosis type 1 were also included in this report.

## 1. Introduction

Several genetic risk factors are known to predispose women to breast cancer. Enhanced screening and preventive mastectomy have been employed to improve the outcome based on the levels of risk [1,2,3,4,5,6]. Breast MRI has repeatedly demonstrated advantages over mammography in detecting tumors in all groups of women, including those carrying germline genetic risk alleles (i.e., mutations, or pathogenic variants—PVs). The tumors detected by MRI tend to be at an earlier stage than those by mammography [4,7,8].

To identify interval cancer between two rounds of annual screening, many high-risk cancer surveillance clinics adopt a schedule of annual mammogram and annual breast MRI, alternating every 6 months in women over the age of 30. In women at elevated risk under age 30, annual mammography is not routinely performed. The health insurance reimbursement schedule also adheres strictly to an interval of 12 months or longer for each screening imaging. When advanced breast screening is first initiated for an individual newly tested positive for a germline high risk PV, some providers attempt to adhere to the schedule aforementioned, start with a baseline mammography or continue the annual mammography, followed by a breast MRI 6 months after the most recent mammography. The recommendation to alternate mammogram and MRI every 6 months came from data generated by a computer simulation model using an assumed cohort in idealist scenario that all *BRCA1* and *BRCA2* mutation women carriers begin screening at age 25 and no women undergo preventive mastectomy, salpingo-oophorectomy or chemoprevention [9,10]. However, the superiority of such practice has not been proven in real life. Many women discovered their genetic high-risk status at a much later age than the recommended to begin MRI screening [11]. For example, for *BRCA1* PV carriers, the first screening breast MRI is recommended to begin at age 25. Regardless of the age to initiate screening, some providers choose to begin mammography followed by MRI 6 months later.

At least nine genes are known to be associated with significantly increased lifetime risk of breast cancer in women: *ATM*, *BRCA1*, *BRCA2*, *CHEK2*, *CDH1*, *PALB2*, *PTEN*, *STK11* and *TP53*. *NF1* PV carriers are also regarded with increased risk between age 30 and 50 years. Yearly screening with mammography and MRI is recommended for these patients [11]. The Moffitt Cancer Center (MCC) GeneHome (GH) clinic functions as a “home” to conduct surveillance, counseling, education, and coordination of care for individuals who carry germline genetic PVs with predisposition to cancer. Individuals tested positive for a PV (pathogenic variant) or LPV (likely pathogenic variant) in a wide spectrum of cancer predisposition genes were referred to GH by providers inside the institution or from the community, including genetic counselors, primary care providers, medical oncologists, oncological surgeons and many other specialists.

Giving the superiority of MRI, we suspect postponing the MRI 6 months later may delay the diagnosis of breast cancer undetectable by mammography. To investigate, we conducted a retrospective chart review in all women visited MCC GH high-risk surveillance clinic for screening. We recorded the age, type of genetic PVs, previous history of cancer, the time of screening imaging and the time of cancer diagnosis.

## 2. Materials and Methods

The screening and surveillance schedule in GH follows the guidelines published by National Comprehensive Cancer Network (NCCN): annual screening mammogram and breast MRI were performed for individuals who carry PVs or LPVs in genes conferring high risk (*BRCA1*, *BRCA2*, *CDH1*, *PALB2*, *PTEN*, *STK11*, and *TP53*) or moderate-high risk (*ATM* and *CHEK2)* of breast cancer. For example, for women carrying *BRCA1* and *BRCA2* PVs, annual bilateral (B/L) breast MRI started at age 25 years old until age 30, then annual B/L screening mammogram was added, alternating or together with annual MRI. For carriers with *TP53* PV, annual MRI started at age 20 until age 30, then annual mammogram was added, alternating or together with MRI. For carriers with *PTEN*, *PALB2* and *CDH1* PV, annual mammogram and MRI both started at age 30. For carriers with *ATM* and *CHEK2* PV, both imaging started at age 40 or earlier if indicated by family history (https://www.nccn.org/professionals/physician_gls/pdf/genetics_bop.pdf (accessed on 1 November 2021) [11]. For carriers with *NF1* PV, both imaging started at age 30. At age 50, annual mammography continued, but MRI was no longer performed. GH clinic coordinates and accommodates the patients’ wishes that imaging be conducted in a preferred diagnostic imaging center, at separate visits or same visit. Depending on the cost or convenience, the patients may choose to have the imaging perform in the cancer center or in a diagnostic center in the community.

For women without a breast risk conferred by genetic PV, they were evaluated for the lifetime risk at the time of initial visit in GH, utilizing the online Tyrer–Cuzick (TC) Model Breast Cancer Risk Evaluation Tool (version 8.0) by International Breast Cancer Intervention Study (IBIS) (https://ibis.ikonopedia.com/, accessed on 1 November 2021) [12]. Following NCCN guidelines, screening mammogram and breast MRI were performed for women whose lifetime breast cancer risk was 20% or higher, even if the genetic PVs they carry are not known to significantly increase the risk of breast cancer to warrant MRI [8,12,13]. Tyrer–Cuzick model was chosen because it is designed to estimates breast cancer risk based on three-generation family history in addition to personal features. Three generation family history is routinely obtained for all GH patients [14].

A chart review was conducted for patients who attended screening and surveillance in MCC GH clinic in a 48-months period from March 2017 to February 2021.

## 3. Results

In this report, two most representative groups of patients were analyzed, women carriers of the breast cancer gene group (*ATM*, *BRCA1*, *BRCA2*, *CHEK2*, *CDH1*, *PALB2*, *PTEN* and *TP53*) and women carriers of mismatch repair (MMR) gene group (*MLH1*, *MSH2*, *MSH6*, *PMS2* and *EPCAM* deletion). Among all individuals cared in GH, 441 women carry at least one PV in the eight genes associated with increased risk for breast cancer (no woman carries *STK11* PV in this group; women with neurofibromatosis type 1 is discussed separately), 26 women carrying PVs in more than one gene were categorized under the gene with the highest breast risk, for example, a woman with PVs in *BRCA2* and *CHEK2* was counted under *BRCA2*, a woman with PVs in *ATM* and *PMS2* was counted under *ATM* (Table 1). Among all, 45.8% (202/441) women have a previous diagnosis of breast cancer, 33.8% (149/441) women underwent bilateral (B/L) mastectomy (prophylactic and therapeutic mastectomy were included), 85.2% (127/149) of which had breast cancer, 66.2% (292/441) women had both or one remaining breast, 25.7% (75/292) of which have a history of breast cancer (for which they underwent lumpectomy or unilateral mastectomy) (Table 2). Ninety-four women carry a PV in five genes associated with MMR Lynch syndrome (Table 3), 21.3% (20/94) have a history of breast cancer, 9.6% (9/94) underwent B/L mastectomy, 100% of which (9/9) had breast cancer, 90.4% (85/94) had both or one remaining breast, 12.9% (11/85) had breast cancer (Table 4).

**Scheme 1:**
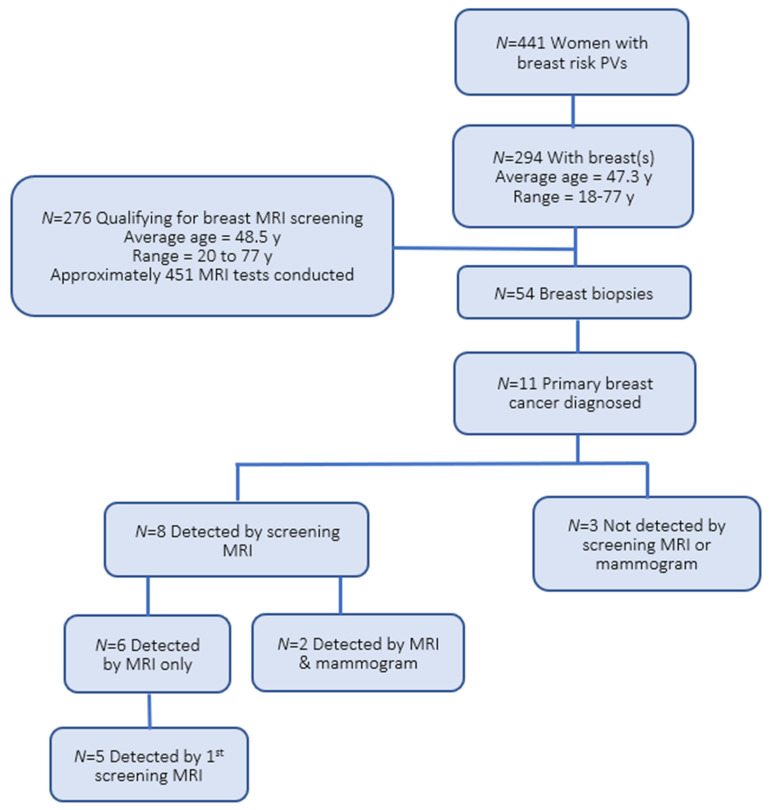
The breast cancer screening and diagnosis in women with increased genetic risk for breast cancer followed in GH.

**Scheme 2:**
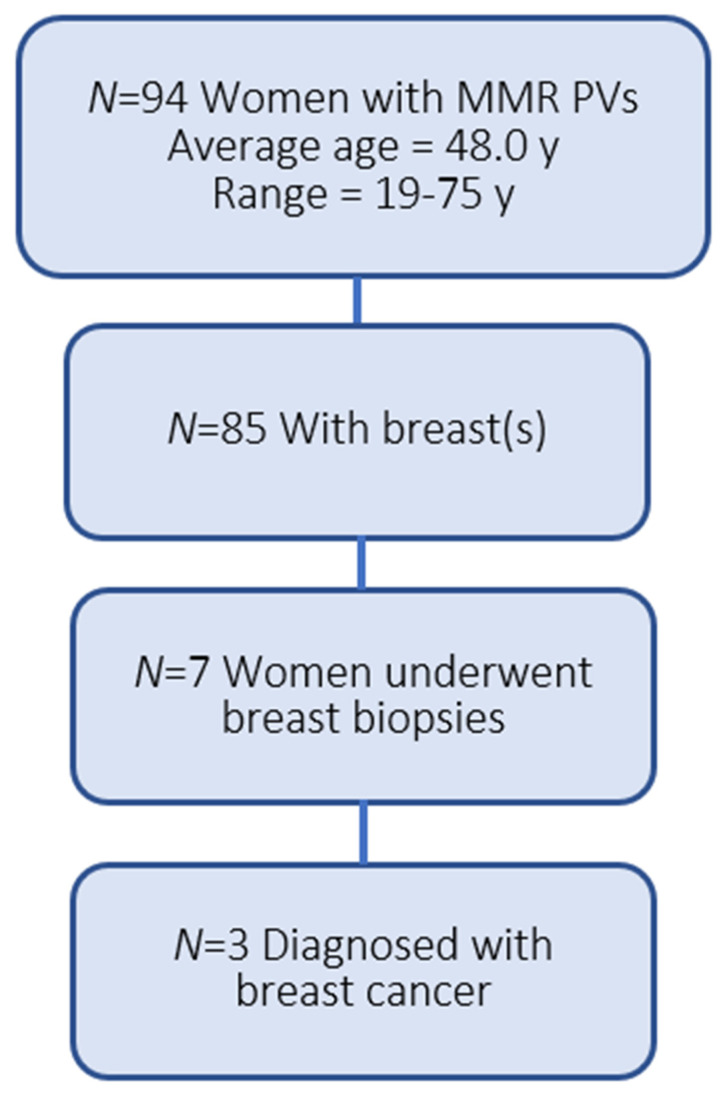
The breast cancer screening and diagnosis in women with MMR gene Lynch syndrome risk followed in GH.

Medical history collected during clinic visits were analyzed (Scheme 1 and Scheme 2). Between 3-2017 and 2-2021, there were 294 female breast risk PV carriers (average age at initial GH visit = 47.3 years, range = 18–77 years), including women who did not qualify for high-risk screening due to their age. Breast MRIs were performed for 276 women in breast risk group who were qualified for MRI screening based on their age, the type of genetic PVs, and with remaining breasts (average age at initial GH visit = 48.5 years, range = 20 to 77 years). MRIs included three categories: screening MRI for high-risk women without symptoms, diagnostic MRI for breasts with suspicious lesions, and surveillance MRI for women with prior history of lumpectomy for breast cancer. These women also received digital breast tomosynthesis and targeted breast ultrasound when indicated. All women had both breasts or one remaining breast after unilateral mastectomy, 27.2% (75/276) of them have had at least one breast cancer diagnosis. The age to begin high-risk screening was based on the type of genes outlined by the NCCN guidelines. Diagnostic imaging was prescribed based on abnormal screening imaging or physical examination. In the following (2nd) year, approximately 62% of the new patients returned to the clinic for high-risk screening. In the 3rd year, 71% to 77% of the patients who presented in the 2nd year returned to the clinic. An estimated 456 breast MRIs were performed in 48 months, average 1.6 tests (range = 1–5 testes) per person. Fifty-four women with breast risk PVs underwent breast biopsy triggered by suspicious breast imaging, including MRI, mammogram and/or ultrasound, 10 underwent biopsy twice, three underwent biopsy thrice. Primary breast cancer was found in 11 women who carry breast risk PVs (Table 5) (Scheme 1), accounted for 3.99% (11/276) of the breast risk group who had one or both breasts and met criteria to undergo high-risk screening, 8 of the 11 (73%) were detectable by breast MRI, six (55%) were detectable by MRI only, two (18%) were detectable by mammogram in addition to MRI (case 7, 8), two were not detectable by MRI but discovered in other fashions (case 9 was a small focus of DCIS incidentally discovered after risk reduction mastectomy, case 10 was a lymph node harboring metastatic breast cancer on the right incidentally discovered by PET/CT surveillance for a left breast cancer undergoing treatment), one was not screened but found incidentally during implants exchange (case 11—this case can be regarded as primary breast cancer because the prior breast cancer was determined as DCIS and B/L mastectomy was performed shortly after the DCIS diagnosis). In all, breast MRI had an estimated detection rate of 1.75% (8/456, equivalent to 18 per 1000 MRI tests) for primary tumor in this cohort of women (Table 5). Five cases were detected by their first screening breast MRI, four of the five were detected within the first month since the initial presentation at GH clinic for high-risk screening. At the time of cancer detection, four women were at least 10 years older than the recommended age to begin screening MRI based on the genetic PVs they carry. One *BRCA1* PV carrier’s invasive breast cancer was diagnosed at age 27, 2 years older than the recommended age to begin screening (age 25).

Three additional recurrent/metastatic breast cancers were palpated in this period of time either by the patient or by the care provider, which accounted for 1.5% (3/202) of the breast risk women who have had a previous diagnosis of breast cancer (Table 5).

Lynch syndrome female PV carriers (average age = 48.0 years, range = 19–75 years) were followed and the breast risk of majority of them were not high enough to warrant MRI screening. Their risk was estimated based on Tyrer–Cuzick model using personal and family history. Ninety percent (85/94) had both breasts or one remaining breast. Seven underwent breast biopsy after abnormal screening imaging in this time period, three primary breast cancers were discovered by routine mammogram screening (3/85, 3.5%) (Table 5) (Scheme 2). Average age at diagnosis was 51.3 years (Median age = 54 years). None of the three women had previous diagnosis of breast cancer and none of them had significantly increased breast cancer risk based on Tyrer–Cuzick model.

In the breast risk PV group (including women who did not qualify for high-risk screening due to their age) (average age = 47.3 years, range = 18–77 years), the breast cancer rate was similar to Lynch syndrome group, 3.4% (10/294). However, the average age at diagnosis in the breast PV group was younger, 46.2 years (Median age = 43 years). In addition, three of the women in breast risk PV group had a previous diagnosis of primary breast cancer (at age 42, 28, and 35 years, respectively, Table 5), one had synchronous bilateral primary breast cancer diagnosed at age 41. Due to the small sample size and limited follow-up time, statistical comparison was not made in the report.

In GH clinic, 50 women have a diagnosis of NF1 (average age = 40.9 years), 6 (12%) of which have had a previous diagnosis of breast cancer, seven underwent breast biopsy, no breast cancer was detected in this period. Nine additional women who carry other genetic mutations underwent breast biopsy. No breast cancer was detected in this period.

## 4. Discussion

Germline genetic pathogenic variants (PVs) in hereditary cancer predisposition genes are responsible for 5% to 10% of diagnosed breast cancers. PVs in *BRCA1* and *BRCA2* possess a 50% to 80% lifetime risk of developing breast cancer [15]. Several other high-risk genes are responsible for lifetime risk of more than 40%: up to 85% for *TP53,* 58% for *PALB2*, 85% for *PTEN*, and 42% for *CDH1* [16,17,18,19]. Based on data from *BRCA1* and *BRCA2* PV carriers, it is accepted that women carrying these high-risk genetic changes will benefit from bilateral preventive mastectomy, even though there are no specific data generated directly from *TP53*, *PALB2*, *PTEN*, or *CDH1* PV carriers [20]. *BRCA1* and *BRCA2* PV carriers are also known to have excessive risk to develop contralateral cancer 20 years after their initial cancer diagnosis [21]. The cumulated risk for the contralateral breast can be as high as 40% [15]. After an occurrence of a unilateral breast cancer, contralateral risk-reduction mastectomy also reduces the cancer incidence of the opposite breast, however, there is insufficient evidence to suggest a survival benefit. For various reasons, many high-risk women decline preventive mastectomy and continue high-risk breast screening [22]. PVs of *ATM* and *CHEK2* confers greater than 30% lifetime risk for breast cancer, but lower than that of *BRCA1* and *BRCA2*, thus are regarded as moderate-high risk. Therefore, risk reduction mastectomy is generally not recommended for *ATM* and *CHEK2* PV carriers [23]. Screening breast MRI has been accepted as the modality of screening for women with greater than 20% lifetime risk [24]. This study investigated the MRI screening and breast cancer detection in a cohort of women carrying high risk and moderate high risk genetic PVs.

Breast MRI has an estimated detection rate of 1.75% (8/456, equivalent to 18 per 1000 MRI tests) for primary tumor in this group of women who carry genetic PVs conferring moderate or high risk for breast cancer. When breaking down into the high risk and moderate risk groups, the estimated rate was 2.28% (23 per 1000 MRI) for *BRCA1* and *BRCA2* group, 2.98% (30 per 1000 MRI) for high risk group (*BRCA1*, *BRCA2*, *CDH1*, *PALB2*, *PTEN*, *TP53*), and 0.83% (8 per 1000 MRI) for moderate risk group (*ATM*, *CHEK2*, *NF1*). These rates of detection are comparable to previously published studies [25,26,27].

Based on the experience of this clinic, we have observed that majority of the individuals were recently tested positive for a genetic breast cancer risk and just began advanced breast screening by MRI at an age that has far exceeded the recommended age to begin MRI. For the high-risk group, the average age initially presented in GH clinic was 48 years (approximately 23% were under 35 years, 44% were 35–55 years, 33% were older than 55 years). Majority of them (approximately 90%) were recently found out they are mutation carriers or made the decision to begin high-risk screening within the past year.

In addition, a number of breast cancer cases (four in five) were diagnosed by their first screening breast MRI within the first month of their initial clinical visit, with three of the four tumors being already at a locally advanced stage. Furthermore, these cancers were not detected by a concurrent or a prior mammography within 1 year preceding the MRI. This finding cautioned the practice that some providers delaying the first screening breast MRI for the purpose of maintaining 6 months interval between a mammography and breast MRI. Breast MRI has an absolute advantage of detecting breast cancer over mammogram. It should be initiated as the first screening modality when a woman is found out to carry a breast risk PV and has passed the age recommended to begin high-risk screening.

Even with advanced and timely screening, we observed breast cancer may still be missed, underscoring the role of risk reduction mastectomy for high-risk PV carriers which has been available as standard care for individuals carrying PVs in *BRCA1*, *BRCA2*, *CDH1*, *PALB2*, *PTEN* and *TP53* (case 8, 9; Table 5) [11]. For example, a minute focus of DCIS (case 8) was discovered by preventive mastectomy 2 months after a non-revealing breast MRI. In another example (case 9), when this woman was undergoing treatment for left breast cancer, a metastatic breast cancer was incidentally discovered in the right axilla’s lymph node by PET/CT scan 7 months after a non-revealing breast MRI of the right breast. No primary site was found in the right breast by mastectomy after neoadjuvant chemotherapy. This metastatic lesion in the lymph node could come from a primary lesion undetectable in the right breast before the neoadjuvant chemotherapy, or from the contralateral primary breast lesion.

In three women who have had unilateral breast cancer and undergone B/L mastectomy, breast palpation examination identified recurrent cancer on the reconstructed breasts (case 11, 12 and 13).

For individuals whose genetic PVs do not significantly increase breast risk, it is equally important to ensure the adherence to routine screening recommended for general population, including screening mammography. Three examples of such cancer were detected by screening mammogram in case 14, 15 and 16. When caring for this high-risk population, we have noticed that neglecting routine cancer screening by the patients is quite common. Underutilizing breast screening is not unique for this population. Based on 2017 United States health statistics, only 67% of the women 50 years and over had a mammogram within the past 2 years [28]. 

Many individuals with genetic risk for multiple cancer types or have had personal or family history of multiple cancers, are often emotionally, physically and financially overwhelmed. They often experience screening fatigue. Many focuses on the type of cancer they perceive as the most dangerous, while neglecting screening for other types which they perceive as not as important. For example, women carrying MMR gene PVs may neglect routine breast cancer screening because it has not been seen in her relatives or it is not listed as an increased risk.

When caring individuals with genetic predisposition to cancers, it is beneficial to have a holistic approach to consider the individual as a whole. In addition to the genetic risk PVs, we should consider risk of all organs, environmental exposures, family histories and any cancer related personal history, such as histology, stage, type of treatment and remission status.

## 5. Conclusions

Women with high-risk genetic predisposition to breast cancer often become aware about the risk at an age much older than the age recommended to begin enhanced screening with MRI. For women carrying high-risk genetic variants, it is likely beneficial to begin breast MRI immediately after the discovery of genetic high-risk, instead of beginning with mammogram, then followed by MRI 6 months later. In addition, despite the extensive screening utilizing MRI, some early-stage breast cancers were missed, highlighting the recommendations to undergo risk reduction mastectomy for women with high-risk PVs.

## Figures and Tables

**Table 1:** Women carrying PVs in breast cancer risk genes.

Genes	*N*=	%
*ATM*	48	11%
*BRCA1*	116	26%
*BRCA2*	136	31%
*CDH1*	7	2%
*CHEK2*	67	15%
*PALB2*	31	7%
*PTEN*	9	2%
*TP53*	24	5%
*BRCA1* + *BRCA2* ^a^	1	
*BRCA2* + *PTEN* ^b^	1	
*ATM* + *CHEK2* ^c^	1	
Total ^d^	441	100%

^a,b,c^ Women carrying PVs in more than one gene. ^d^ Twenty women carrying PVs in more than one gene were categorized under the gene with highest breast risk.

**Table 2:** History of breast cancer and mastectomy in women carrying PVs in eight breast cancer genes.

	*N*=	%
Breast cancer and B/L mastectomy	127	29%
No breast cancer and B/L mastectomy	22	5%
Breast cancer	75	17%
No breast cancer	217	49%
Total	441	100%

**Table 3:** Women carrying PVs in MMR Lynch syndrome genes.

Genes	*N*=	%
*MLH1*	13	14%
*MSH2*	26	28%
*MSH6*	20	21%
*PMS2*	34	36%
*EPCAM* deletion	1	1%
Total	94	100%

**Table 4:** History of breast cancer and mastectomy in women carrying PVs in Lynch syndrome genes.

	*N*=	%
Breast cancer and B/L mastectomy	9	9%
No breast cancer and B/L mastectomy	0	0%
Breast cancer	11	12%
No breast cancer	74	79%
Total	94	100%

**Table 5:** Malignant and premalignant breast lesions discovered during GeneHome follow up.

ID	Age at Diagnosis	Caner Type (Stage)	Method of Detection	Germline PV	Prior Cancer Hx (Age) and Surgery (Age)	Time Since Initial GH Visit/Hx of Breast Imaging
1	54	DCIS, TisN0	Screening MRI, non-mass enhancement.	*BRCA1*	None	<1 month; detected by 1st screening MRI. MA (BI-RADS 1) 10 months ago.
2	58	IDC, T1aN0	Screening MRI, oval mass.	*ATM*	Thyroid cancer (47 y), invasive papillary, plus minimally invasive follicular type; dermatofibroma/dermatofibrosarcoma protuberans (55 y); total thyroidectomy (47 y)	<1 month; detected by 1st screening MRI. MA (BI-RADS 0) 11 months ago, left breast nodular asymmetry led to US: complex cysts needing short interval follow up.
3	43	ILC, T3N0	Screening MRI, non-mass enhancement.	*CDH1*	Cervical cancer (28 y); hysterectomy (36 y)	1 month; detected by 1st screening MRI. MA (BI-RADS 3) 3 weeks ago.
4	61	DCIS, microinvasive	Screening MRI, non-mass enhancement.	*BRCA1*	Multiple cutaneous basal cell carcinoma and squamous cell carcinoma.	11 months; detected by 1st screening breast MRI at age 61; screening MA (BI-RADS 2) 5 months ago.
5	34	IDC, pT1bN0M0	Screening MRI; oval mass.	*TP53* VUS ^a^	None.	2.5 years; detected by screening MRI; MA (BI-RADS 2) 5 months ago; MRI (BI-RADS 1) 12 months ago.
6	66	IMC, pT1aN0M0	Screening MRI; irregular mass.	*BRCA1*	TLH/BSO (age 66).	1.5 years; detected by screening MRI; MA (BI-RADS 1) 10 months ago; MRI (BI-RADS 1) 16 months ago.
7	27	IDC, pT1aN0M0	Screening MRI and 3D MA; non-mass enhancement on MRI, irregular hypoechoic mass with internal vascularity on MA.	*BRCA1*	None	<1 month; detected by 1st screening MRI and 1st screening 3D MA at the same time.
8	41	IMC (Lt, T1cN1(mi)); DCIS (Rt, pTisN0)	Palpable nodules on right; multi-focal bilateral lesions were identified by diagnostic tomosynthesis MA, MRI and US	*PTEN*	Cerebella dysplastic gangliocytoma, s/p resection (age 40); goiter.	Masses palpated by physical exam at initial GH visit; detectable by diagnostic MA; no prior screening imaging.
9	58	DCIS	Incidental finding in right breast during risk reduction B/L mastectomy.	*BRCA2*	Left breast locally invasive IDC (42 y); B/L breast reduction (38 y)	1 month; revealed by mastectomy; benign screening breast MRI 2 months ago.
10	29	Metastatic breast cancer in Rt axilla lymph node	Incidental finding: PET/CT surveillance for left breast node positive IDC found FDG avid lymph node in right axilla (it is possibly a second primary cancer).	*BRCA1*	Left breast IDC, node positive (left) cT3cN1M0 (28 y).	9 months; metastatic lesion in LN detected by PET/CT 7 months after negative finding in breast MRI 9 months ago and MA 1 month ago. No breast primary was found on the right. side
11	38	Possible 2nd primary IDC, T1N0M0	Incidental finding on implant capsule when undergoing implants exchange.	*TP53, BRIP1*	DCIS identified incidentally (35 y) during breast reduction surgery (35 y); s/p bilateral mastectomy at age 35.	3.5 years.
12	55	Local metastatic breast cancer	Self-reported skin change.	*BRCA2*	Breast cancer, 1st primary (37 y); local recurrence (42 y); B/L mastectomy (42 y); TLH/BSO (45 y).	9 months; self-reported skin change.
13	45	Recurrent breast cancer (Lt), node positive IDC	Provider-palpated nodule on left reconstructed breast mound.	*BRCA2*	Breast DCIS, 2 loci microinvasive (Lt, 39 y), B/L mastectomy (40 y); BSO (41 y)	9 months; palpated 5 years after the mastectomy and reconstruction.
14	44	Recurrent breast cancer (Lt)	Provider-palpated nodule on left reconstructed breast mound.	*CHEK2* ^b^	Breast IDC (pT1cpN1a, left, 39 y); B/L mastectomy (39 y) papillary thyroid ca (1 cm) in an ovarian stroma without lesion in thyroid gland (T1N0M0, 41 y); USO (41 y)	24 months; palpated 2 years after mastectomy 2 years ago and implants replacement 3 months ago.
15	54	IDC	Screening 3D MA; structural asymmetry.	*MSH6*	Cutaneous basal cell carcinoma on skin, TLH/BSO (52 y)	12 months; benign MA 18 months ago.
16	46	DCIS, TisN0,	Screening 3D MA, calcifications.	*PMS2*	Thyroid cancer (43 y), papillary type; melanoma in situ on scalp (44 y); total thyroidectomy (43 y); hysterectomy (39 y)	10 months; previously benign yearly MA.
17	54	ILC, cT1cN0M0	Self-palpated mass.	*EPCAM* deletion	Colon cancer (age 45); colectomy; hysterectomy (40 y); BSO (45 y)	Self-palpated prior to the initial GH visit; previously benign yearly MA.

MRI: magnetic resonance imaging; MA: Mammogram; US: ultrasound; PET/CT: positron emission tomography-computed tomography; DCIS: ductal carcinoma in situ; IDC: invasive ductal carcinoma; ILC: invasive lobular carcinoma; IMC: invasive mammary carcinoma; ADH: typical ductal hyperplasia; ALH: atypical lobular hyperplasia; LN: lymph node; Lt: left; Rt: right. TLH: total laparoscopic hysterectomy; BSO: bilateral salpingo-oophorectomy; USO: unilateral salpingo-oophorectomy; B/L mastectomy: bilateral mastectomy. ^a^ *TP53* variant of uncertain significance (VUS), highly suspicious for pathogenicity (this variant tracks with cancer in six individuals and three generations in this family). ^b^ This *CHEK2* c.470T > C (p.Ile157Thr) is a common variant believed to have lower cancer risk penetrance than classical *CHEK2* mutation.

## Data Availability

The data presented in this study are available from the corresponding author upon request.
